# Domestication affects sex-biased gene expression evolution in the duck

**DOI:** 10.1098/rsos.221313

**Published:** 2023-04-05

**Authors:** Hongchang Gu, Liang Wang, Xueze Lv, Weifang Yang, Li Zhang, Zebin Zhang, Tao Zhu, Yaxiong Jia, Yu Chen, Lujiang Qu

**Affiliations:** ^1^ Institute of Animal Husbandry and Veterinary Medicine, Beijing Academy of Agriculture and Forestry Sciences, Beijing, People's Republic of China; ^2^ Department of Animal Genetics and Breeding, National Engineering Laboratory for Animal Breeding, College of Animal Science and Technology, China Agricultural University, Beijing, People's Republic of China; ^3^ Beijing Municipal General Station of Animal Science, Beijing, People's Republic of China; ^4^ Evolutionary Biology Centre, Uppsala University, Uppsala, Sweden; ^5^ Institute of Animal Science, Chinese Academy of Agricultural Sciences, Beijing, People's Republic of China

**Keywords:** sex-biased expression, sequence evolution, sexual selection, domestication, duck

## Abstract

Genes with sex-biased expression are thought to underlie sexually dimorphic phenotypes and are therefore subject to different selection pressures in males and females. Many authors have proposed that sexual conflict leads to the evolution of sex-biased expression, which allows males and females to reach separate phenotypic and fitness optima. The selection pressures associated with domestication may cause changes in population architectures and mating systems, which in turn can alter their direction and strength. We compared sex-biased expression and genetic signatures in wild and domestic ducks (*Anas platyrhynchos*), and observed changes of sexual selection and identified the genomic divergence affected by selection forces. The extent of sex-biased expression in both sexes is positively correlated with the level of both d*_N_*/d*_S_* and nucleotide diversity. This observed changing pattern may mainly be owing to relaxed genetic constraints. We also demonstrate a clear link between domestication and sex-biased evolutionary rate in a comparative framework. Decreased polymorphism and evolutionary rate in domesticated populations generally matched life-history phenotypes known to experience artificial selection. Taken together, our work suggests the important implications of domestication in sex-biased evolution and the roles of artificial selection and sexual selection for shaping the diversity and evolutionary rate of the genome.

## 1. Introduction

In most sexually reproducing animals, evolutionary conflicts of interest arise whenever males and females interact and their routes to fitness maximization differ, often termed sexual conflict [1–4]. When this conflict revolves around which individuals enjoy priority mating rights, how many offspring are produced, when they are produced, and how much each parent invests into these offspring, sexual selection has also begun to play a role in sexual evolution [5–7]. Sexual selection is selected for traits of one sex, and therefore may be a decisive force in shaping sexual dimorphism [8,9]. We can understand many sexual evolutionary phenomena in a closed-loop way, sexual conflict is the driving force behind sexual selection, and sexual selection shapes phenotypic sexual dimorphism and expression bias, ultimately resolving conflicts of interest between males and females. As mentioned above, sex-biased genes allow each sex to reach separate optima. However, genetic constraints, as a ‘hinderance’, often prevent the resolution of sexual conflict, that is, this evolutionary force seems to be trying to reduce sexual dimorphism, and it also seems to be affecting the sequence characteristics and evolutionary pattern of the genome [10,11]. Concretely, such constraints are generally considered to be related to the diversity and evolution of sex-biased expression, because the biased expression is thought to reduce constraints and thereby enable rapid adaptive evolution and more variation [12,13].

Clearly, a tug-of-war involving many evolutionary factors shapes the unique genetic properties of biased genes. There is no evidence that a directional link exists between sexual selection and sequence evolution, however, proteins encoded by sex-biased genes do show greater amino acid sequence divergence [14,15]. It is foreseeable that as the degree of sex bias (fold change; FC) becomes more extreme, the intensity of sexual selection will increase, while the trend of constraints is the opposite. The existence of genetic signatures such as changes in genetic diversity is the result of a trade-off of multiple evolutionary forces, which means that it is challenging to analyse each evolutionary force independently. When we try to research the populations that already exist in nature, because of the time spans that allow us to observe evolutionary effects, the problem seems to be on the verge of being solved. Domestication has the potential to greatly alter sexual conflict and sexual selection via an altered mating system, and we might expect this to quickly affect sex-biased gene expression and associated population genomic characteristics [16–18]. Both monogamy (having one mate) and polygamy (having several mates) exist in mallard (*Anas platyrhynchos*), and mate choice is ultimately up to the female. This mating system results in male–male competition for mating opportunities and thus leads to more intense sexual selection in wild populations, although it also exists among females [19,20], males are subjected to strong sexual selection [21]. Duck domestication occurred initially roughly 2200 years ago, and controlled breeding by humans has weakened or eliminated male–male competition, female choice and sperm competition, thereby leading to relaxed sexual selection compared to wild populations [16]. During domestication, genes are expected to experience both high-intensity artificial selection and relaxed sexual selection, it is hard but relative roles and the genetic signatures of these selective forces is yet to be untangled [22].

We chose male and female RNA-seq data from gonadal and liver tissue from one wild breed (mallard duck, MD), one meat-type breed (Pekin duck, PK) and one egg-type breed (Gaoyou duck, GY) to assess the effects of domestication on expression at the early development stage. Both the two domesticated breeds, GY and PK, have experienced a common domestication event, but subsequently they underwent varying sex-specific selection regimens, allowing us to observe the evolutionary effects of artificial selection. These two varying sex-specific selection regimens are caused by artificial selection for different purposes. Many duck breeds (like GY) have been selected explicitly for increased female fecundity, resulting in these breeds that produce numerous and large eggs. Therefore, these breeds result from elevated female-specific selection compared with the mallard ancestor. On the contrary, the meat-type duck (like PK) is artificially selected to be larger in size, more muscular and faster in growth, which corresponds to a stronger male-specific selection compared with the mallard ancestor. Populations that have experienced domestication were selected in order to identify whether the magnitude of sexual selection is associated with sex-biased evolution. Leaning on detailed information on sequence diversity and evolutionary rate, we have determined the evolutionary effect of shaping genome signature intra-breeds and during domestication. Our results provide a clear link between sex-biased gene evolution and domestication through comprehensive population genetic analysis.

## 2. Results

### 2.1. Changes of sexual size dimorphism owing to domestication

**Figure 1.**
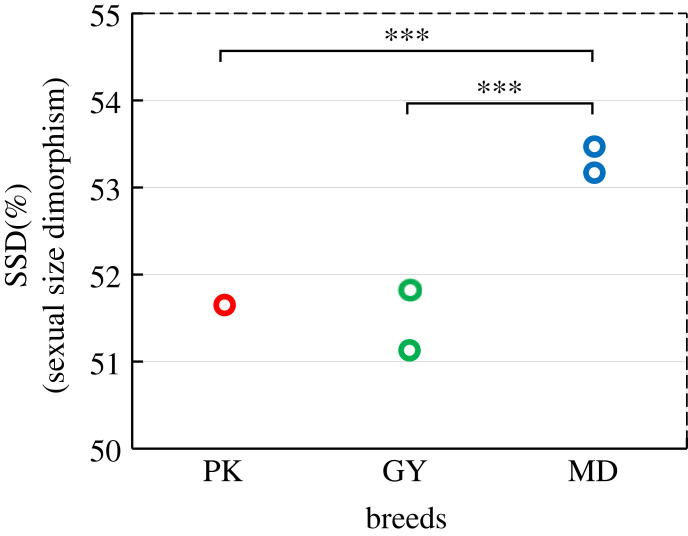
Sexual size dimorphism (SSD) is associated with domestication in ducks. dimorphism in ducks. SSD is calculated as the ratio between the average body weight of males compared to the total average weight (males and females). *Z*-tests for two independent samples were used so that individual weight data and sample size were both taken into consideration. Significant difference between SSD of three breeds is indicated (*Z*-test, *p < 0.05, **p < 0.01, ***p < 0.001).

The extent of the sexual size dimorphism (SSD) is thought to be an indicator of sexual selection [23,24], and we, therefore, assessed the potential changes in phenotypic dimorphism associated with domestication. We calculated the SSD parameter of the three breeds (figure 1), and the magnitude of SSD correlates with mean body weight, which is one of the most striking examples of sex differences in animals [25]. Although there are two sets of data from different sources for GY and MD (figure 1; electronic supplementary material, table S3), after a one-to-one test, there are significant SSD differences between the two domestic duck populations and the wild population (PK: 51.65%, GY: 51.13% and 51.82%, MD: 53.17% and 53.47%, *p* = 0.00067, *Z*-test). Specifically, MD show more significant male-skewed SSD even though males are larger than females in all observed breeds.

### 2.2. Sex-biased expression on the autosomes

We quantified the extent of the sex-biased gene expression in two different tissues (gonad and liver) collected at the embryonic stage (table 1; electronic supplementary material, figure S1), focusing on autosomal loci owing to the complexity of incomplete Z chromosome dosage compensation in birds. We defined sex-bias as greater than 2 FC and a significant difference in expression between the sexes (false discovery rate (FDR) < 0.05). As expected, the gonad showed a higher proportion of sex-biased genes compared to the liver. We observed large differences in the direction of sex-biased expression in the gonad of domesticates, possibly owing to significant differences in sex-specific selection between egg-type and meat-type ducks (*Z*-test, *p* = 0.032). Specifically, PK shows a greater proportion of male-biased genes (*n* = 901, 57% of sex-biased genes), while female-biased genes are more prevalent in GY (*n* = 1506, 65% of sex-biased genes). For the liver of all three breeds, less than 5% of genes showed a significant sex bias, even in the two domesticated breeds, this proportion is less than 1%. Wild populations showed a significantly greater level of sex-biased expression compared to domestics for both male-biased and female-biased genes (Mann Whitney *U*-test, *p* < 0.05) (electronic supplementary material, figure S2).

**Table 1.** Description of sex-biased gene expression in the gonad and liver.

tissue	no. of genes expressed	no. of sex-biased gene of MD		no. of sex-biased gene of GY		no. of sex-biased gene of PK	
		total, males, females	proportion (%)	total, males, females	proportion (%)	total, males, females	proportion (%)
gonad	11 033	2583 1330 1253	23.41	2316 810 1506	20.99	1566 901 665	14.19
liver	9189	389 160 229	4.23	67 26 39	0.73	85 32 53	0.93

**Figure 2.**
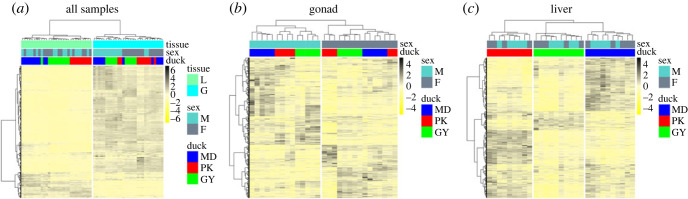
Heatmaps and hierarchical clustering of gene expression for (*a*) all samples; (*b*) gonad; and (*c*) liver. Shown is the average relative expression for all autosomal expressed genes from two tissues (L, liver; G, gonad) of M (male) and F (female) in three breeds (MD, PK and GY).

**Figure 3.**
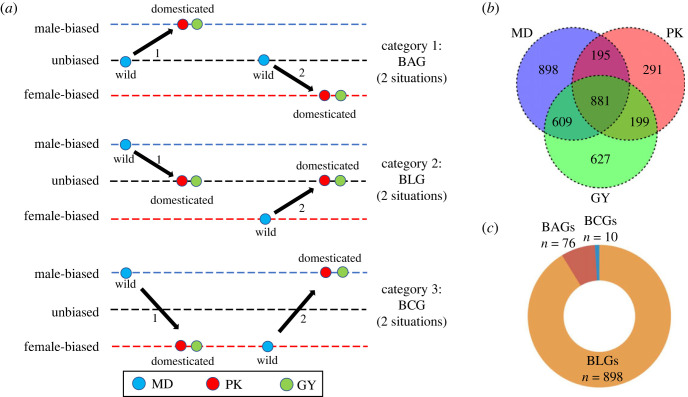
Dynamic changes of sex-biased genes in three breeds. (*a*) Three categories of sex-biased expression changes among wild duck and domesticated duck. (*b*) A Venn diagram illustrating statistical results of sex-biased expression in three breeds. (*c*) The three categories of sex-biased changes and their numbers and proportions.

We next used hierarchical clustering of expression levels for all samples of two tissues (figure 2). All samples are clustered by tissue, sex-biased expression was most evident in the gonad, where samples largely cluster first by sex, in contrast to liver samples which clustered first by breed, and there were 881 sex-biased genes in all breeds (figure 3*b*). Genes that are sex-biased in MD but unbiased in both domestic ducks are termed as bias-lost genes (BLGs) (figure 3*a*). We observed 898 BLGs in the gonad (figure 3*b,c*). We observed 76 bias-acquired genes (BAGs), which are unbiased in MD but sex-biased in both domestic ducks, and 10 bias-converted genes (BCGs), which are sex-biased in both three breeds but reverse the direction in domestic ducks (figure 3*a,c*).

For the top 10 gene ontology (GO) terms of male-biased pattern, biological processes involved in reproduction, muscle development and embryonic development were identified (electronic supplementary material, table S1). Genes that were universally female-biased were enriched for regulatory processes of reproduction and fear response (electronic supplementary material, table S1). For bias-lost expression, genes were enriched for behaviour and morphogenesis, which could be indicative of relaxed sexual selection (electronic supplementary material, table S2).

### 2.3. Coding sequence evolution during domestication

**Figure 4.**
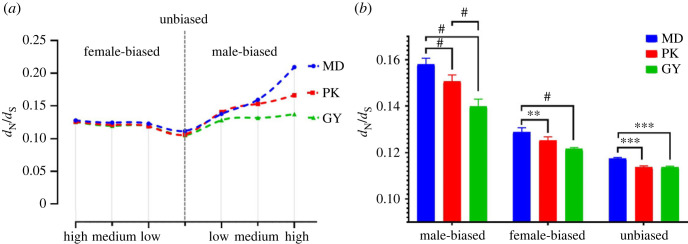
Average ratio of nonsynonymous substitutions (*d*_N_) to synonymous substitutions (*d*_S_) for male-biased, female-biased and unbiased genes in three breeds. (*a*) Relationship between *d*_N_/*d*_S_ and extent of sex-biased expression in gonads of three breeds, ‘high’, ‘medium’ and ‘low’ below the *x*-axis are shorthand for ‘high biased expression’, ‘medium biased expression’ and ‘low biased expression’, respectively. (*b*) Differences in *d*_N_/*d*_S_ among breeds for three biased categories. Displayed significance scores are *p < 0.05, **p < 0.01 and ***p < 0.001.

To explore the impact of domestication on sequence evolution rate, and determine whether the overall pattern of selection is distinct in different sex-biased categories, we visualized the relationship between mean *d*_N_/*d*_S_ and the extent of sex-biased expression in gonads of three breeds for male-biased and female-biased genes (figure 4*a*) [26,27]. We observed that the mean *d*_N_/*d*_S_ of sex-biased genes, especially male-biased genes, was higher within breeds than those genes with unbiased expression. We also observed a strong positive relationship between the evolutionary rate and the level of male bias, that is highly male-biased genes show greater divergence than lowly male-biased genes and female-biased genes. Female-biased genes have a flatter rise in evolutionary speed than male-biased genes.

Among three breeds, all genes in MD universally had higher *d*_N_/*d*_S_ levels than those in two domesticated breeds (figure 4*b*). Strongly male-biased genes show an elevated *d*_N_/*d*_S_ between breeds, and this divergence of evolution rate has been maximized (MD, 0.2093; PK, 0.1661; GY, 0.1409) when FC is greater than 10 (high male-biased expression). Interestingly, with the increase of male directional FC, the difference in the rate of sequence evolution between the two domesticated groups becomes more obvious. Male-biased genes of all three categories in PK show a higher evolutionary rate than GY, this may be related to relaxed purifying selection or enhanced positive selection.

We also evaluated the coding sequence evolution of BLGs to understand the effects of loss or reduced sexual conflicts following domestication. The *d*_N_/*d*_S_ ratio of BLGs in GY was statistically significantly lower than MD and PK, while the difference between the latter two is not significant (*p* = 0.19; electronic supplementary material, figure S3).

### 2.4. Sequence polymorphism of three breeds

**Figure 5.**
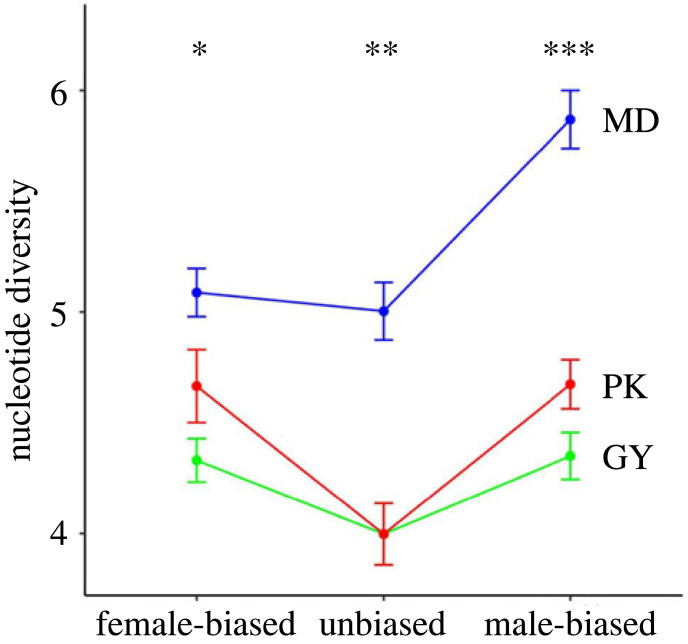
Amount of genetic diversity in 10 kb windows explained by the nucleotide diversity (π) for female-biased, unbiased, and male-biased genes expressed in gonads of three breeds. Genetic diversity also significantly varied across three breeds in the same bias categories (Wilcoxon Rank sum test, *p < 0.05, **p < 0.01, ***p < 0.001).

The nucleotide diversity (π), was used as a measure of polymorphism, to reflect the imprint of selection force on sequence diversity before and after domestication (figure 5) [28,29]. Given there are too few sex-biased genes in the liver, and the gonads are subject to strong sexual selection through sperm competition, we assessed the extent of the sequence diversity using the average π value corresponding to different categories of sex-biased genes based on 10 kb sliding windows only in the gonad. Among all sex-biased expression patterns among three breeds, genetic diversity was significantly higher in MD. Specifically, the nucleotide diversity for sex-biased genes was significantly higher than unbiased genes, and this was particularly pronounced for male-biased genes. Interestingly, the two domesticated breeds maintain a high degree of consistency in sequence polymorphism. This low genetic diversity in two domesticated populations highlights the importance of artificial selection in affecting sequence variation.

**Figure 6.**
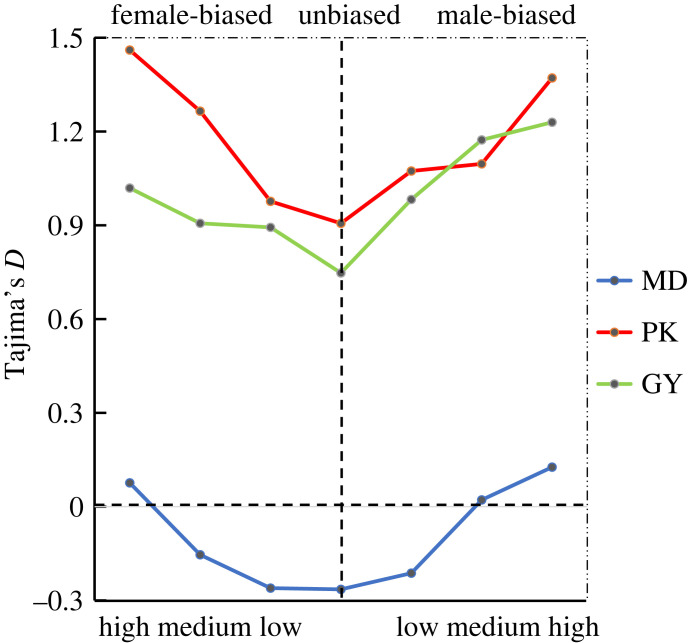
Relationship between Tajima's *D* and extent of sex-biased expression in gonads of MD, PK and GY, ‘high’, ‘medium’ and ‘low’ below the *x*-axis are shorthand for ‘high biased expression’, ‘medium biased expression’ and ‘low biased expression’, respectively.

Finally, we assessed Tajima's *D* for sex-biased genes [30], which summarizes the site-frequency spectrum and reflects several potential evolutionary forces (figure 6). We observe elevated Tajima's *D* for both male- and female-biased genes across all breeds, although genome-wide estimates vary markedly across breeds.

## 3. Discussion

Selection for domestication results in extreme evolutionary pressures, and changes in the mating system, sperm competition and mate choice in domesticates have the potential to vastly shape sex-specific selection. We assessed the potential for domestication to affect sex-specific selection within the genome by assessing the changes in sex-biased gene expression at the embryonic stage, and its sequence properties, in one wild and two domestic duck breeds. Consistent with phenotypic changes in SSD (figure 1), we observe reduced overall proportions of sex-biased genes in the gonad in domestics compared to wild ancestors (table 1). The gonad, as a reproductive tissue, has a higher number of sex-biased genes than the liver as a somatic tissue in all three breeds. This result is consistent with previous research, and can be explained by the fact that the gonads show much greater sexual dimorphism than other tissues [31–33], the result also reflects the decrease of gene expression diversity during domestication events, this effect seems to be common in animals and plants [34]. Our samples were taken from embryonic birds, and the amount of sex-biased gene expression tends to increase during development, with low levels at embryonic stages and high levels in sexually mature adults. Previous studies on birds indicate that 35%∼50% of genes show sex-biased expression in adult gonad which is higher than the ratio in our study (less than 25%) [35,36]. We insisted on used RNA-seq data obtained from the embryonic day 25 gonad for two key reasons. First, we expected to remove interference from acquired factors and environmental effects. Second, female-specific selection in birds is strongest during this developmental time point [35].

The earliest artificial selection may have been unconscious, and subsequent selection drove the emergence of the entire domestication process [37,38]. In addition to purposeful selection, inbreeding of populations and changes in the mating system also occur during and after domestication, and the latter has led to relaxed sexual selection [39,40]. The reduced proportion of sex-biased genes in domestics may result from the removal or reduction in sexual selection. Our results are somewhat different than previous work in *Drosophila* which has shown that both males and females exhibit transcriptional feminization after the removal of sexual selection [41]. It is not clear whether this discordance reflects fundamental differences in selective pressures, or is a consequence of differences in study design. For example, the *Drosophila* work was focused on adult samples while our research was based on embryonic tissues [42], and there are major differences through development in the proportion, direction and evolutionary signatures of sex-biased genes in both birds [35] and *Drosophila* [43].

We estimated the ratio of the nonsynonymous to the synonymous substitution rate (*d*_N_/*d*_S_) to measure the rate of sequence evolution. Like many previous studies [44–47], we observe obviously elevated rates of sequence evolution in male-biased genes, and, to a lesser extent, the female-biased evolutionary rate also increased compared to unbiased genes (figure 4*a,b*), and this pattern is similar and clear across all three breeds. Many sex-biased genes, especially the extremely biased ones, often correspond to sex-specific phenotypes or functions and also show narrower expression patterns, so these genes tend to evolve under more sexual selection and less constraint than unbiased genes. If other evolutionary effects are stable, elevated sexual selection increases the rate at which genetic diversity is lost, while relaxed constraint shows faster variant accumulation [48–50]. Thus, it is possible to infer the efficacy of these forces through nucleotide diversity intra-breed. Our intra-breed results suggest that the power of relaxed constraint rather than sexual selection is a major driver that sex-biased genes accumulate variation faster than genes with unbiased expression. Notably, both protein sequence evolutionary rate and genetic diversity of unbiased genes and biased genes differ significantly between the three breeds, this pattern is not breed-specific. Artificial selection will lead to the result that domesticated populations often show low diversity [37], apparently, this force is not just on sex-biased genes. These inter-breed molecular hallmarks suggest that domestication plays a major role in shaping genome diversity and sequence evolution via artificial selection compared with other bias-related selection power (sexual selection and genetic constraint).

Although the inseparable relationship between domestication and relaxed sexual selection is entirely reasonable at the theoretical level, we still found phenotype evidence to prove this crucial link. As an indicator of the intensity of sexual selection, SSD is usually reflected by the male/female weight ratios, and for most birds, including ducks, males are larger on average than females [51]. Our results consistently show the same SSD pattern in which the male is heavier. However, the body size differentiation between the sexes seems to be more extreme in the wild duck population (SSD ratio = 53.14%, *p* = 0.00067). Assimilation of body size among sexes in domesticated populations shows that the sexual selection maintained by male–male competition or a special mating system is weakened after domestication, which is consistent with our analysis that relaxed sexual selection does exist during domestication. It is worth noting that an effective population ratio between sexes can also reflect sexual selection intensity [52]. Previous studies about the effective sex ratio of mallard pointed to results that males outnumber females [53,54]. The limited supply of females and the resulting competition for mates are the root cause of strong sexual selection in mallards. Predictably, because of domestication and subsequent artificial insemination, this male-skewed mode would decrease and even reverse.

Owing to the consistency of natural conditions and evolutionary processes, the differences in Tajima's *D* signify the diverse effect of many selection forces including sexual selection. Normally, Tajima's *D* = 0 under neutrality while *D* > 0 indicates balancing selection to maintain multiple variants and *D* < 0 purifying selection or a recent selective sweep [47]. According to the value of Tajima's *D*, we observed that the category and extent of sex-biased genes determine the net effect of several selection processes. Specifically, female-biased genes with an elevated *D* show signs of balancing selection or relaxed purifying selection within populations, the homogeneity trend observed in male-biased genes may also indicate similar inferences. An intriguing observation was that the MD population corresponds to a lower level of Tajima's *D* compared with GY and PK, we had sufficient reasons to infer that this case was not caused by relaxed sexual selection because it also seemed to exist in the whole genome rather than sex-biased genes. This is somewhat surprising that in addition to strongly biased genes, MD showed negative average values for Tajima's *D*, and this result was contradictory to the high genetic diversity of mallard breeds. This ‘illogicality’ also exists in domesticated ducks whose average Tajima's *D* is always more than 0.5. This genome-wide extraordinary Tajima's *D* results may be explained by the difference in the effective population size (*N*_e_) [55], which is much greater in MD than in GY and PK owing to the strong genetic bottleneck associated with domestication. This bottleneck leads to a sudden decrease of *N*_e_ in domesticated ducks, and the callback effect of subsequent population expansion events was weak [56]. Another reason may be artificial balancing selection that exists in order to pursue heterosis during the selection of local duck breeds.

The non-adaptive genetic drift, an evolutionary force that cannot be ignored during domestication, makes a fast evolutionary rate at extremely male-biased genes. Previous research conjectured that genetic drift rather than sexual selection promoted sex-biased sequence evolution [14]. In this research, the effect of genetic drift or codon usage bias may be secondary. The faster sequence evolution rate is mainly achieved by the increase of the non-synonymous mutation rate rather than the decrease of the synonymous mutation rate. Given our results and the fact that sexual selection underlies sexual dimorphism, sexual selection and the constraint do affect sequence evolution. Compared with genetic drift and natural selection, sexual selection is directional and only acts on sex-biased genes. Our results indicate that the effect of relaxed sexual selection to shape genomic diversity is counteracted and even reversed by artificial selection during domestication inter-breed. Although our research has determined the role of sexual selection and artificial selection during domestication, several evolutionary forces apparently could push the populations towards a similar direction in this process, which was reflected by the genetic architecture in the genome or transcriptome. For instance, genetic drift and artificial selection can also lead to a decreased level of genetic diversity, while populations subject to balancing selection will show an increase in diversity. Thereby, accurately identifying the effects of a certain evolutionary force using a single detection indicator is hard to achieve. It can be foreseen that these larger datasets, more ideal experimental models, and genetic indicators with multi-parameter and multi-omics, are essential to interpret the complex patterns of evolutionary power and natural variation.

Compared with millions of years of natural selection, short-term domestication has only been thousands of years, since the emergence of human civilization. However, the impact of human intervention on the natural evolution of wild populations is far-reaching. On the one hand, directional selection and small-group captive breeding have reduced biodiversity. On the other hand, the loss of free mating rights has caused the original mating system to be overthrown and rebuilt, which also affects sexual dimorphism and biodiversity. To conclude, we used a combination of a wild breed and broad-time-scale animal models that have undergone a domestication event, to disentangle the evolutionary power that can change genome architecture. Genome-wide evidences proved that although the relaxed sexual selection is not a negligible causal factor for shaping sex-biased diversity, artificial selection mainly explains these signatures of the genome. In addition, evolutionary events such as founder effects, drift, and mixed ancestry undeniably occur during domestication, but equally predictably, these effects are negligible compared to the high-intensity effect of artificial purposeful modification and selection, do not affect our outcome orientation. Our results also implicate that standing genetic variation and sequence evolution within populations represent the outcome of several interacting processes, and in this scenario, domestication and genetic constraints as powerful forces in shaping these special genomic patterns of sex-biased genes under different population dimensions. Although sample size limitations limit the evidence to support only early developmental stages of sex-biased expression, combining similar studies and conjectures, our conclusions should be ‘generally applicable’ throughout the developmental stages of the organism.

## 4. Material and methods

### 4.1. Sample collection and sequencing

We obtained fertilized eggs from two domesticated duck breeds. These ducks include one meat-type breed, PK; and one egg-type breed, GY. We also obtained fertilized eggs of MD as wild ducks. The classification of production types follows the description of Animal Genetic Resources in China Poultry. All eggs were kept under standard incubator conditions, qualified eggs were selected by observation of vascular distribution at the early embryonic stage and periodic weighing. For each breed, the left gonad and liver were dissected separately from five male and five female individuals in the laboratory setting where there is an incubator and immediately placed in RNAlater (Invitrogen, Carlsbad, CA, USA) at 25 embryonic days (ed25). At this stage, the duck embryo has developed to the eve of hatching, the liver and many organs have developed to the level of 0 days old, and the gonad is in the rapid development stage. The female-specific selection is strong during this developmental time point [57,58]. RNA was prepared with the Animal Tissue RNA Kit (Qiagen, Hilden, Germany). All tissue samples were sequenced using Illumina NovaSeq 6000 (Illumina Inc., San Diego, CA, USA) as paired-end 150 bp reads at 10× coverage, yielding a total of 685.02 Gb of paired-end reads.

Twenty-four adult individuals of three populations (PK, GY, MD; *n* = 8) were used to collect whole blood from the brachial veins of ducks by standard venipuncture. The three groups came from three provinces in China separated by more than 1000 km, of which PK came from Beijing, GY came from Jiangsu province, and MD came from Ningxia province. Genomic DNA was extracted using the standard phenol/chloroform method. We sequenced each sample at 5× depth in order to reduce the false-negative rate of variants owing to our strict filter criteria. We randomly selected one individual for 10× coverage, except for the MD, where we sequenced seven individuals at 5 coverage and a random one at 20× coverage. Finally, we generated 159 Gb of paired-end reads. We assessed the quality of both re-sequencing data and RNA-seq data and conducted filtering with fastp v. 0.20. using default parameters.

### 4.2. RNA-seq and data processing

RNA-seq high-quality reads were mapped to the reference genome of *A. platyrhynchos* (GenBank Accession *GCA_003850225.1*) [59] using Hisat2 v. 2.1.0 [60,61]. Transcript abundances for the annotated genes were estimated using StringTie v. 2.1.2 [62,63], where relative expressions are expressed as fragments per kilobase of exon per million mapped reads (FPKM) values. Genes with FPKM of less than 0.5 in all samples were considered not expressed and would be deleted. We deleted all sex chromosome genes because the sex-biased expression is defined based on autosomes. In addition, we also deleted all immune-related genes and the major histocompatibility complex gene family located on duck micro chromosome 17, because these genes have some potential confounding effects independent of sexual conflict [64–66].

### 4.3. Hierarchical clustering and heatmaps

Hierarchical clustering was performed using the pvclust package [67] of R, with bootstrap resampling (1000 replicates) using Euclidean clustering with complete linkage. Heatmaps were separately generated for all samples, liver and left gonad using log2 average expression of male and female autosomal genes using the R package pheatmap v. 1.0.12.

### 4.4. Sex-biased gene expression

Normalized FPKM expression counts of autosomal genes for each sex were used to calculate sex bias, with fold-change ratios between males and females, starting at unlogged twofold, and a significance threshold of *p* < 0.05 after adjusting for multiple testing. The sex-biased genes were further divided into four categories based on fold FC: low bias (2–4 FC), medium bias (4–10 FC) and high bias (greater than 10 FC), resulting in three categories for male-biased genes, three categories for female-biased genes, and an unbiased gene category (less than 2 FC). To further determine the dynamic changes of sex-biased expression, we also identified three classes of genes of autosomal, BLGs, that is, genes that show biased expression in wild breeds are lost in both two domesticated breeds. BAGs refer to genes that only show sex-biased expression in domesticated breeds. BCGs reflect the reversal of sex-biased expression patterns before and after domestication.

### 4.5. Estimation of sexual size dimorphism

We measured the phenotypes of body weight of 50 (25 males and 25 females) healthy adult PK (12 weeks old) obtained from Beijing Golden Duck Co., Ltd (Beijing, China). Detailed weight data of adult MD and GY are collected from previous studies [68–70], sample information is presented in the electronic supplementary material, table S3.

SSD refers to the differences between males and females of the same species, such as in body size, and weight, and is critical for a better understanding of the dynamic changes of sexual selection. Body size was the mean body mass in grams of adult males and adult females [71], and we calculated (adult male weight/total weight) per cent to visualize SSD, *Z*-tests for two independent samples were used to test whether the SSD is significantly different between three breeds.

### 4.6. Population genomics analysis

To assess the polymorphism and sequence evolutionary characteristics that may be caused by dynamic changes in the long-term effects of sexual selection on domestication, we calculated multiple population genetic parameters in the three breeds separately, including both the sex-biased genes and unbiased genes. Compared with the liver and other tissues, the gonads are more sexually dimorphic, and more phenotypic dimorphic tissues within the body exhibit greater levels of transcriptional dimorphism. We therefore only focused on sex-biased genes in the gonad for the next analysis. Nucleotide diversity was used as a measure of sequence diversity. We extract the corresponding gene sets from the reference genome of *A. platyrhynchos* according to seven sex-biased gene categories (including the unbiased genes). Nucleotide sequence for autosomal genes of three breeds was mapped to the corresponding gene sets using BWA v. 0.7.17 [72]. Picard Tools v. 2.26.0 was used to convert the mapping file from Sam to Bam format. Duplicate reads were removed from individual sample alignments using MarkDuplicates. The Genome Analysis Toolkit v. 4.2.1.0 (GATK) RealignerTargetCreator, and IndelRealigner protocol were used for global realignment of reads around INDELs [73]. We filtered variants both per breed and per individual using GATK according to the stringent filtering criteria. Finally, we obtained polymorphism results under the 10 kb window through VCFtools v. 0.1.13 [74]. The statistical data corresponding to the tail end of genes truncated by 10 kb windows was removed, and then the average of π was extracted. This approach avoids the problem of uncertainty of window statistics' results, especially sex-biased genes that exist discretely in the genome.

Variation in standing genetic variation within populations represents the outcome of several interacting processes, notably balancing selection and purifying selection. To estimate the net effect of these processes, we estimated Tajima's *D* [30] using VCFtools v. 0.1.13 for sex-biased genes in three breeds.

To further examine the potential explanatory power of relaxed sexual selection resulting from domestication, we extracted the ratio of the non-synonymous to the synonymous substitution rate (*d*_N_/*d*_S_). *Gallus Gallus*, as an outgroup, was used to find the orthologous genes. Sex-biased genes would be identified that gene symbols are exactly the same, and these genes also satisfy reciprocal top hits from a BLASTn with an e-value cutoff of 1×10^−10^ and a minimum percentage identity of 60%. We identified variants of biased and unbiased genes using the previous method, Python scripts were written to calculate *d*_N_/*d*_S_ using VCF files. Specifically, we used the sequencing data of each individual to compare to the reference genome and then get the VCF file. We used the mutation information in the VCF file to deduce the base sequence of each individual target gene by our python script (https://github.com/zhutao1009/dnds), and finally used the aligned fasta sequence file of these genes to calculate the dN/dS of the sequence. The outliers that only appeared in a single breed were removed, and then the average value of *d*_N_/*d*_S_ was calculated.

### 4.7. Gene ontology

In order to determine the certain biological functions and pathways corresponding to bias-lost genes (BLGs) and sex-biased genes, we conducted a GO enrichment analysis using the clusterProfiler package [75] of R. Genes that have a universal bias pattern across all individuals are regarded as high-confidence male-bias or female-biased genes. To make the enrichment results more meaningful, we use the database human (*Homo sapiens*) rather than chicken (*G. gallus*) for annotation. Enrichment was determined using an FDR-corrected significance threshold of 0.05.

## Data Availability

Whole-genome resequencing data used in this study are available on the NCBI short read archive (Accession PRJNA419832). RNA-seq reads have been deposited in NCBI Sequence Read Archive under accession numbers SRP269397. Data is also available in the electronic supplementary material [76].

## References

[1] Dapper AL, Wade MJ. 2016 The evolution of sperm competition genes: the effect of mating system on levels of genetic variation within and between species. Evolution **70**, 502-511. (10.1111/evo.12848) 26748568 PMC4868060

[2] Kasimatis KR, Nelson TC, Phillips PC. 2017 Genomic signatures of sexual conflict. J. Hered. **108**, 780-790. (10.1093/jhered/esx080) 29036624 PMC5892400

[3] Parsch J, Ellegren H. 2013 The evolutionary causes and consequences of sex-biased gene expression. Nat. Rev. Genet. **14**, 83-87. (10.1038/nrg3376) 23329110

[4] Rowe L, Chenoweth SF, Agrawal AF. 2018 The genomics of sexual conflict. Am. Nat. **192**, 274-286. (10.1086/698198) 30016158

[5] Andersson M, Iwasa Y. 1996 Sexual selection. Trends Ecol. Evol. **11**, 53-58. (10.1016/0169-5347(96)81042-1) 21237761

[6] Hosken DJ, House CM. 2011 Sexual selection. Curr. Biol. **21**, R62-R65. (10.1016/j.cub.2010.11.053) 21256434

[7] Kokko H, Brooks R, Jennions MD, Morley J. 2003 The evolution of mate choice and mating biases. Proc. R. Soc. B **270**, 653-664. (10.1098/rspb.2002.2235) PMC1691281 12769467

[8] Read AF, Harvey PH. 1989 Validity of sexual selection in birds - reply. Nature **340**, 105. (10.1038/340105a0)

[9] Wong BBM, Candolin U. 2005 How is female mate choice affected by male competition? Biol. Rev. **80**, 559-571. (10.1017/s1464793105006809) 16221329

[10] Lande R. 1980 Sexual dimorphism, sexual selection, and adaptation in polygenic characters. Evolution **34**, 292-305. (10.2307/2407393) 28563426

[11] Rice WR. 1984 Sex-chromosomes and the evolution of sexual dimorphism. Evolution **38**, 735-742. (10.2307/2408385) 28555827

[12] Assis R, Zhou Q, Bachtrog D. 2012 Sex-biased transcriptome evolution in *Drosophila*. Genome Biol. Evol. **4**, 1189-1200. (10.1093/gbe/evs093) 23097318 PMC3514954

[13] Leichty AR, Pfennig DW, Jones CD, Pfennig KS. 2012 Relaxed genetic constraint is ancestral to the evolution of phenotypic plasticity. Integr. Comp. Biol. **52**, 16-30. (10.1093/icb/ics049) 22526866 PMC3381942

[14] Harrison PW, Wright AE, Zimmer F, Dean R, Montgomery SH, Pointer MA, Mank JE. 2015 Sexual selection drives evolution and rapid turnover of male gene expression. Proc. Natl Acad. Sci. USA **112**, 4393-4398. (10.1073/pnas.1501339112) 25831521 PMC4394296

[15] Zhang Y, Sturgill D, Parisi M, Kumar S, Oliver B. 2007 Constraint and turnover in sex-biased gene expression in the genus *Drosophila*. Nature **450**, 233-237. (10.1038/nature06323) 17994089 PMC2386141

[16] Gering E, Incorvaia D, Henriksen R, Wright D, Getty T. 2019 Maladaptation in feral and domesticated animals. Evol. Appl. **12**, 1274-1286. (10.1111/eva.12784) 31417614 PMC6691326

[17] Driscoll CA, Macdonald DW, O'Brien SJ. 2009 From wild animals to domestic pets, an evolutionary view of domestication. Proc. Natl Acad. Sci. USA **106**, 9971-9978. (10.1073/pnas.0901586106) 19528637 PMC2702791

[18] Chen J, Ni P, Li X, Han J, Jakovlic I, Zhang C, Zhao S. 2018 Population size may shape the accumulation of functional mutations following domestication. BMC Evol. Biol. **18**, 4. (10.1186/s12862-018-1120-6) 29351740 PMC5775542

[19] Birkhead TR, Atkin L, Moller AP. 1987 Copulation behavior of birds. Behaviour **101**, 101-138. (https://www.jstor.org/stable/4534592)

[20] Birkhead TR, Pizzari T. 2002 Postcopulatory sexual selection. Nat. Rev. Genet. **3**, 262-273. (10.1038/nrg774) 11967551

[21] Collet JM, Dean RF, Worley K, Richardson DS, Pizzari T. 2014 The measure and significance of Bateman's principles. Proc. R. Soc. B **281**, 20132973. (10.1098/rspb.2013.2973) PMC3973258 24648220

[22] Wright D, Rubin CJ, Barrio AM, Schutz K, Kerje S, Brandstrom H, Kindmark A, Jensen P, Andersson L. 2010 The genetic architecture of domestication in the chicken: effects of pleiotropy and linkage. Mol. Ecol. **19**, 5140-5156. (10.1111/j.1365-294X.2010.04882.x) 21040053

[23] Dale J, Dunn PO, Figuerola J, Lislevand T, Szekely T, Whittingham LA. 2007 Sexual selection explains Rensch's rule of allometry for sexual size dimorphism. Proc. R. Soc. B **274**, 2971-2979. (10.1098/rspb.2007.1043) PMC2211517 17878139

[24] Soulsbury CD, Kervinen M, Lebigre C. 2014 Sexual size dimorphism and the strength of sexual selection in mammals and birds. Evol. Ecol. Res. **16**, 63-76.

[25] Szekely T, Freckleton RP, Reynolds JD. 2004 Sexual selection explains Rensch's rule of size dimorphism in shorebirds. Proc. Natl Acad. Sci. USA **101**, 12 224-12 227. (10.1073/pnas.0404503101) PMC514460 15304645

[26] Bolivar P, Mugal CF, Nater A, Ellegren H. 2016 Recombination rate variation modulates gene sequence evolution mainly via GC-biased gene conversion, not Hill-Robertson interference, in an avian system. Mol. Biol. Evol. **33**, 216-227. (10.1093/molbev/msv214) 26446902 PMC4693978

[27] Ellegren H, Parsch J. 2007 The evolution of sex-biased genes and sex-biased gene expression. Nat. Rev. Genet. **8**, 689-698. (10.1038/nrg2167) 17680007

[28] Ellegren H, Galtier N. 2016 Determinants of genetic diversity. Nat. Rev. Genet. **17**, 422-433. (10.1038/nrg.2016.58) 27265362

[29] Nei M, Li WH. 1979 Mathematical-model for studying genetic-variation in terms of restriction endonucleases. Proc. Natl Acad. Sci. USA **76**, 5269-5273. (10.1073/pnas.76.10.5269) 291943 PMC413122

[30] Tajima F. 1989 Statistical-method for testing the neutral mutation hypothesis by DNA polymorphism. Genetics **123**, 585-595. 2513255 10.1093/genetics/123.3.585 PMC1203831

[31] Goldman TD, Arbeitman MN. 2007 Genomic and functional studies of *Drosophila* sex hierarchy regulated gene expression in adult head and nervous system tissues. PLoS Genet. **3**, 2278-2295. (10.1371/journal.pgen.0030216) PMC2082469 18039034

[32] Mank JE, Hultin-Rosenberg L, Webster MT, Ellegren H. 2008 The unique genomic properties of sex-biased genes: insights from avian microarray data. BMC Genomics **9**, 1-4. (10.1186/1471-2164-9-148) 18377635 PMC2294128

[33] Parisi M, Nuttall R, Naiman D, Bouffard G, Malley J, Andrews J, Eastman S, Oliver B. 2003 Paucity of genes on the *Drosophila* X chromosome showing male-biased expression. Science **299**, 697-700. (10.1126/science.1079190) 12511656 PMC1363366

[34] Liu W et al. 2019 Decrease of gene expression diversity during domestication of animals and plants. BMC Evol. Biol. **19**, 19. (10.1186/s12862-018-1340-9) 30634914 PMC6330456

[35] Mank JE, Nam K, Brunstrom B, Ellegren H. 2010 Ontogenetic complexity of sexual dimorphism and sex-specific selection. Mol. Biol. Evol. **27**, 1570-1578. (10.1093/molbev/msq042) 20142440

[36] Pointer MA, Harrison PW, Wright AE, Mank JE. 2013 Masculinization of gene expression is associated with exaggeration of male sexual dimorphism. PLoS Genet. **9**, e1003697. (10.1371/journal.pgen.1003697) 23966876 PMC3744414

[37] Diamond J. 2002 Evolution, consequences and future of plant and animal domestication. Nature **418**, 700-707. (10.1038/nature01019) 12167878

[38] Conner JK. 2003 Artificial selection: a powerful tool for ecologists. Ecology **84**, 1650-1660. (10.1890/0012-9658(2003)084[1650:Asaptf]2.0.Co;2)

[39] Dapper AL, Wade MJ. 2020 Relaxed selection and the rapid evolution of reproductive genes. Trends Genet. **36**, 640-649. (10.1016/j.tig.2020.06.014) 32713599

[40] Swanson WJ, Vacquier VD. 2002 The rapid evolution of reproductive proteins. Nat. Rev. Genet. **3**, 137-144. (10.1038/nrg733) 11836507

[41] Hollis B, Houle D, Yan Z, Kawecki TJ, Keller L. 2014 Evolution under monogamy feminizes gene expression in *Drosophila melanogaster*. Nat. Commun. **5**, 3482. (10.1038/ncomms4482) 24637641

[42] Mank JE, Ellegren H. 2009 All dosage compensation is local: gene-by-gene regulation of sex-biased expression on the chicken Z chromosome. Heredity **102**, 312-320. (10.1038/hdy.2008.116) 18985062

[43] Perry JC, Harrison PW, Mank JE. 2014 The ontogeny and evolution of sex-biased gene expression in *Drosophila melanogaster*. Mol. Biol. Evol. **31**, 1206-1219. (10.1093/molbev/msu072) 24526011 PMC3995337

[44] Campos JL, Johnston KJA, Charlesworth B. 2018 The effects of sex-biased gene expression and x-linkage on rates of sequence evolution in *Drosophila*. Mol. Biol. Evol. **35**, 655-665. (10.1093/molbev/msx317) 29228267

[45] Dutoit L, Mugal CF, Bolivar P, Wang M, Nadachowska-Brzysk K, Smeds L, Yazdi HP, Gustafsson L, Ellegren H. 2018 Sex-biased gene expression, sexual antagonism and levels of genetic diversity in the collared flycatcher (*Ficedula albicollis*) genome. Mol. Ecol. **27**, 3572-3581. (10.1111/mec.14789) 30055065

[46] Grath S, Parsch J. 2012 Rate of amino acid substitution is influenced by the degree and conservation of male-biased transcription over 50 Myr of *Drosophila* evolution. Genome Biol. Evol. **4**, 346-359. (10.1093/gbe/evs012) 22321769 PMC3318448

[47] Sayadi A, Barrio AM, Immonen E, Dainat J, Berger D, Tellgren-Roth C, Nystedt B, Arnqvist G. 2019 The genomic footprint of sexual conflict. Nat. Ecol. Evol. **3**, 1725-1730. (10.1038/s41559-019-1041-9) 31740847

[48] Huang HT, Rabosky DL. 2015 Sex-linked genomic variation and its relationship to avian plumage dichromatism and sexual selection. BMC Evol. Biol. **15**, 10. (10.1186/s12862-015-0480-4) 26377432 PMC4574164

[49] Lao O, Kayser M. 2008 Mechanisms and effects of natural selection. Med. Gen. **20**, 308-314. (10.1007/s11825-008-0122-y)

[50] Weinreich DM, Watson RA, Chao L. 2005 Perspective: sign epistasis and genetic constraint on evolutionary trajectories. Evolution **59**, 1165-1174. 16050094

[51] Ancona S, Liker A, Cristina Carmona-Isunza M, Szekely T. 2020 Sex differences in age-to-maturation relate to sexual selection and adult sex ratios in birds. Evol. Lett. **4**, 44-53. (10.1002/evl3.156) 32055410 PMC7006465

[52] Ancona S, Denes FV, Krueger O, Szekely T, Beissinger SR. 2017 Estimating adult sex ratios in nature. Phil. Trans. R. Soc. B **372**, 20160313. (10.1098/rstb.2016.0313) 28760756 PMC5540855

[53] Humburg DD, Prince HH, Bishop RA. 1978 Social-organization of a mallard population in northern Iowa. J. Wildl. Manage. **42**, 72-80. (10.2307/3800691)

[54] Ohde BR, Bishop RA, Dinsmore JJ. 1983 Mallard reproduction in relation to sex-ratios. J. Wildl. Manage. **47**, 118-126. (10.2307/3808058)

[55] Wiberg RAW, Veltsos P, Snook RR, Ritchie MG. 2021 Experimental evolution supports signatures of sexual selection in genomic divergence. Evol. Lett. **5**, 214-229. (10.1002/evl3.220) 34136270 PMC8190450

[56] Zhang Z et al. 2018 Whole-genome resequencing reveals signatures of selection and timing of duck domestication. Gigascience **7**, giy027. (10.1093/gigascience/giy027) 29635409 PMC6007426

[57] Li S, Bai S, Qin X, Zhang J, Irwin DM, Zhang S, Wang Z. 2019 Comparison of whole embryonic development in the duck (*Anas platyrhynchos*) and goose (*Anser cygnoides*) with the chicken (*Gallus gallus*). Poult. Sci. **98**, 3278-3291. (10.3382/ps/pez133) 30941418

[58] Moghadam HK, Pointer MA, Wright AE, Berlin S, Mank JE. 2012 W chromosome expression responds to female-specific selection. Proc. Natl Acad. Sci. USA **109**, 8207-8211. (10.1073/pnas.1202721109) 22570496 PMC3361381

[59] Zhou Z et al. 2018 An intercross population study reveals genes associated with body size and plumage color in ducks. Nat. Commun. **9**, 2648. (10.1038/s41467-018-04868-4) 30018292 PMC6050300

[60] Kim D, Langmead B, Salzberg SL. 2015 HISAT: a fast spliced aligner with low memory requirements. Nat. Methods **12**, 357-360. (10.1038/nmeth.3317) 25751142 PMC4655817

[61] Kim D, Paggi JM, Park C, Bennett C, Salzberg SL. 2019 Graph-based genome alignment and genotyping with HISAT2 and HISAT-genotype. Nat. Biotechnol. **37**, 907-915. (10.1038/s41587-019-0201-4) 31375807 PMC7605509

[62] Pertea M, Pertea GM, Antonescu CM, Chang T-C, Mendell JT, Salzberg SL. 2015 StringTie enables improved reconstruction of a transcriptome from RNA-seq reads. Nat. Biotechnol. **33**, 290-295. (10.1038/nbt.3122) 25690850 PMC4643835

[63] Pertea M, Kim D, Pertea GM, Leek JT, Salzberg SL. 2016 Transcript-level expression analysis of RNA-seq experiments with HISAT, StringTie and Ballgown. Nat. Protoc. **11**, 1650-1667. (10.1038/nprot.2016.095) 27560171 PMC5032908

[64] Ghosh R, Andersen EC, Shapiro JA, Gerke JP, Kruglyak L. 2012 Natural variation in a chloride channel subunit confers avermectin resistance in *C. elegans*. Science **335**, 574-578. (10.1126/science.1214318) 22301316 PMC3273849

[65] Hedrick PW. 2011 Population genetics of malaria resistance in humans. Heredity **107**, 283-304. (10.1038/hdy.2011.16) 21427751 PMC3182497

[66] Stahl EA, Dwyer G, Mauricio R, Kreitman M, Bergelson J. 1999 Dynamics of disease resistance polymorphism at the Rpm1 locus of *Arabidopsis*. Nature **400**, 667-671. 10458161 10.1038/23260

[67] Suzuki R, Shimodaira H. 2006 Pvclust: an R package for assessing the uncertainty in hierarchical clustering. Bioinformatics **22**, 1540-1542. (10.1093/bioinformatics/btl117) 16595560

[68] Boos M, Zorn T, Le Maho Y, Groscolas R, Robin JP. 2002 Sex differences in body composition of wintering mallards (*Anas platyrhynchos*): possible implications for survival and reproductive performance. Bird Study **49**, 212-218. (10.1080/00063650209461268)

[69] Janiszewski P, Murawska D, Hanzal V, Gesek M, Michalik D, Zawacka M. 2018 Carcass characteristics, meat quality, and fatty acid composition of wild-living mallards (*Anas* *platyrhynchos* L.). Poult. Sci. **97**, 709-715. (10.3382/ps/pex335) 29211883

[70] Zhu C, Xu W, Tao Z, Liu H, Song W, Zhang S, Yuan Z, Li H. 2020 Effects of rearing in cages on slaughter performance and intestinal microbiota of Gaoyou ducks (*Anas platyrhynchos*). J. Agri. Biotechnol. **28**, 2200-2208.

[71] Greenwood JG. 2003 Measuring sexual size dimorphism in birds. Ibis **145**, E124-E126. (10.1046/j.1474-919X.2003.00175.x)

[72] Li H, Durbin R. 2009 Fast and accurate short read alignment with Burrows-Wheeler transform. Bioinformatics **25**, 1754-1760. (10.1093/bioinformatics/btp324) 19451168 PMC2705234

[73] McKenna A et al. 2010 The genome analysis toolkit: a MapReduce framework for analyzing next-generation DNA sequencing data. Genome Res. **20**, 1297-1303. (10.1101/gr.107524.110) 20644199 PMC2928508

[74] Danecek P et al. 2011 The variant call format and VCFtools. Bioinformatics **27**, 2156-2158. (10.1093/bioinformatics/btr330) 21653522 PMC3137218

[75] Yu G, Wang L-G, Han Y, He Q-Y. 2012 clusterProfiler: an R Package for comparing biological themes among gene clusters. Omics-J. Integr. Biol. **16**, 284-287. (10.1089/omi.2011.0118) PMC3339379 22455463

[76] Gu H et al. 2023 Domestication affects sex-biased gene expression evolution in the duck. Figshare. (10.6084/m9.figshare.c.6533391)

